# Searching for the bottom of the ego well: failure to uncover ego depletion in Many Labs 3

**DOI:** 10.1098/rsos.180390

**Published:** 2018-08-08

**Authors:** Miguel A. Vadillo, Natalie Gold, Magda Osman

**Affiliations:** 1 Departamento de Psicología Básica, Universidad Autónoma de Madrid, Madrid, Spain; 2 Department of Philosophy, University of Oxford, Oxford, UK; 3 School of Biological and Chemical Sciences, Queen Mary University of London, London, UK

**Keywords:** ego depletion, impossible anagram task, Many Labs 3, persistence, self-control, Stroop

## Abstract

According to a popular model of self-control, willpower depends on a limited resource that can be depleted when we perform a task demanding self-control. This theory has been put to the test in hundreds of experiments showing that completing a task that demands high self-control usually hinders performance in any secondary task that subsequently taxes self-control. Over the last 5 years, the reliability of the empirical evidence supporting this model has been questioned. In the present study, we reanalysed data from a large-scale study—Many Labs 3—to test whether performing a depleting task has any effect on a secondary task that also relies on self-control. Although we used a large sample of more than 2000 participants for our analyses, we did not find any significant evidence of ego depletion: persistence on an anagram-solving task (a typical measure of self-control) was not affected by previous completion of a Stroop task (a typical depleting task in this literature). Our results suggest that either ego depletion is not a real effect or, alternatively, persistence in anagram solving may not be an optimal measure to test it.

## 1. Introduction

According to a popular model of self-control, willpower is in many ways analogous to a muscle [1,2]. Just as a muscle gets tired, our willpower is temporarily exhausted by tasks demanding the inhibition of impulses, so that little energy remains available for future acts demanding self-control. This hypothesis has been put to the test in hundreds of laboratory experiments revealing that participants' performance in a task that requires them to override an irrelevant dominant response is significantly poorer if prior to it they completed a different task requiring self-control [3]. Since the seminal publication of this effect, known as *ego depletion* [4,5], it has been replicated numerous times. However, over the last 5 years, the reliability of the ego depletion effect has become the subject of heated debate [6]. In this paper, we present a study that adjudicates this debate.

A comprehensive meta-analysis by Hagger *et al*. [3] revealed a medium-to-large average effect size for these studies, but further meta-analyses and failed attempts to replicate ego depletion have cast the effect into doubt. A reanalysis of the studies originally meta-analysed by Hagger *et al*. [3] revealed that this literature might be strongly biased by the selective publication of statistically significant findings or by data dredging and significance chasing [7], a suspicion confirmed by an independent series of meta-analyses published shortly afterwards [8] (but see also [9]). In response, Hagger *et al*. [10] coordinated a large-scale registered replication report (RRR) conducted simultaneously in 23 different laboratories using a common protocol. The results were unsettling: ego depletion reached statistical significance in only two laboratories, yielding an average effect size indistinguishable from zero.

Commenting on this outcome, Baumeister & Vohs [11] argued that the RRR relied on a poorly chosen depleting task. Hagger *et al*. [10] presented participants with a series of words on a computer screen and asked them to press a button if they saw an ‘e’ unless the ‘e’ was next to or one letter away from another vowel. In the no depletion condition, a key was pressed whenever an ‘e’ was present. Baumeister & Vohs pointed out that in order to induce ego depletion, there would usually be a prior stage, carried out by all participants, which would establish a habitual, dominant response, so that the depletion condition would involve overcoming a prior impulse. Thus, it is unlikely that Hagger *et al*.'s depletion task would reduce performance on a second self-control task because participants were not all given a preliminary habit-forming task of pressing a computer key whenever they saw an ‘e’, which they would have to overcome in the depletion condition. In support of this interpretation, an independent reanalysis of the RRR data conducted by Dang [12] found that the ego depletion effect was in fact significant within a subset of participants who stated that they had invested more effort on the e-crossing task. However, it is not totally clear whether this feature of the study can account for failing to observe ego depletion in the RRR, given that other studies (including the seminal study on which the RRR was based [13]) have reported positive results without using a preliminary habit-forming period (see also [14]).

The goal of the present study was to offer a new, high-powered test of ego depletion effects by reanalysing a pre-existing dataset from a recent large-scale multi-site study: Many Labs 3 [15]. The primary goal of the multi-site study was to explore whether university students' performance in a range of cognitive and personality-based tasks degrades across the academic term due to self-selection, whereby the more conscientious students participate at the beginning of term, compared to the students who wait until the last minute. Most importantly, for our present purposes, they all performed two tasks that are frequently employed in ego depletion research, namely a Stroop task and an impossible anagram-solving task (see the Procedure and design section for details of these and the other tasks). These conveniently fulfil the conditions that Baumeister & Vohs [11] outline. The Stroop has been used extensively as a depleting task in ego depletion studies with positive results [3,9]. It is the canonical example of a task requiring the inhibition of a dominant response. Persistence in anagram-solving tasks has also been a common dependent measure in ego depletion studies [8]. Based on this literature, we can ‘postdict’ that the amount of time that participants in Many Labs 3 spent solving the anagrams should depend on whether they had completed a Stroop task previously or not: participants who were asked to solve the anagrams after completing a Stroop task should spend less time on them than participants who were asked to solve the anagrams before doing a Stroop task. In the present study, we put this hypothesis to the test.

## 2. Participants and methods

### 2.1. Participants

The original study comprised 3433 participants. All the tasks and tests they performed were sorted randomly across a sequence of slots (see details in the Procedure and design section). For the reasons outlined below, only participants who completed the anagram-solving task between slots 6 and 22 were eligible for the present reanalysis. Consequently, the following analyses are based on a sample of 2062 participants.

### 2.2. Procedure and design

The original study comprised 10 effects, 10 individual difference measures, three data quality indicators, and a selection of demographic items. Among others, the protocol included personality assessment, Stroop, metaphoric structuring, word availability, persistence, conscientiousness, power and perspectives task, embodiment task, self-esteem task and a moral judgement task (see [15] for full details). For our purposes, only two of the tasks are relevant: the Stroop [16] and the persistence task [17].

In the Stroop task, participants are essentially asked to perform an inhibition task, in which they respond to the font colour of a series of words presented on the screen while ignoring the semantics of the words, which are themselves names of colours. The Stroop effect consists of participants showing longer reaction times when the font colours and the colour names are incongruent than when they are congruent. In Many Labs 3, the Stroop task comprised 12 training trials followed by 63 testing trials. At both stages, the words and the colour of the fonts were congruent in one-third (25/75) of the trials and incongruent in the remaining two-thirds (50/75) of the trials. Although this number of trials is relatively small compared to other studies that have used the Stroop as a depleting task, which may have hundreds of trials (e.g. [18]), there is evidence of depletion effects with only 20 incongruent trials [19]. According to the results reported in Many Labs 3 [15], the size of the Stroop effect was somewhat smaller (*d* = 0.91) than previous demonstrations of the effect [16], possibly due to the use of a smaller number of trials and also a higher proportion of incongruent trials, but it was nevertheless quite robust (statistically significant in all subsamples), showing that participants did experience difficulties with inhibiting the dominant response.

Persistence was measured using the unsolvable anagram task, where participants are presented with a series of anagrams, some of them unsolvable. Participants can choose how long they spend solving them, which is taken as an index of persistence. In Many Labs 3, the protocol moved automatically to the next task after a fixed period of 240 s, if participants had not moved on already, which limits the length of time that participants could persist. Among the 2062 participants included in the present analyses, 366 had a score of 240 regarding this variable, giving rise to a bimodal distribution. To ensure that their inclusion in the analyses did not bias the results (e.g. because of their impact in the distribution of the dependent variable), all the analyses carried out were separately conducted with and without these participants. Given that the results were virtually identical in both cases, for the sake of simplicity in the Results section, we report only the results of the whole sample, although, admittedly, this violates the normality assumption of the regression tests.

As explained above, the full protocol comprised several effects and measures collected in a single 30 min session. The experimental session was divided into 26 pages or slots and participants completed 10 tasks in semi-random order across those slots. Roughly half performed the anagram-solving task before the Stroop task and roughly half completed the tasks in the opposite order. We will refer to the former as condition anagram first (AF) and the latter as condition Stroop first (SF). Our main hypothesis is that participants in the SF condition persisted less in the anagram-solving task than participants in the AF condition. A potential confound between these two conditions is that, because the experimental tasks were presented in random order, participants in the SF condition tended, on average, to complete the anagram-solving task later in the experimental session than participants in the AF condition. To control for this confound, for each participant, we recorded when (i.e. in which slot) they completed the anagram task within the experimental session and we included this as a possible predictor in our analyses. Additionally, to reduce the collinearity between condition and slot, we removed from the sample all participants who completed the anagram task before slot 6 (because at that point in the session, they could only belong to condition AF) or after slot 22 (because at that point in the session, they could only belong to condition SF). Most importantly, the results reported below do not depend on this exclusion criterion, although for the sake of simplicity, we only report the results of the analyses excluding these participants.

It is worth noting that there are alternative ways to operationalize task order in the protocol of Many Labs 3. For instance, as the experimental session included 10 studies spanning 26 pages or slots, it would be possible to use study order (1–10) instead of slot order (1–26) in the statistical analyses. However, we felt that slot order provided a more precise control for study duration and fatigue effects, given that study order ignores the fact that some studies were longer and spanned several slots, while others were briefer and comprised a single slot.

## 3. Results

**Figure 1.**
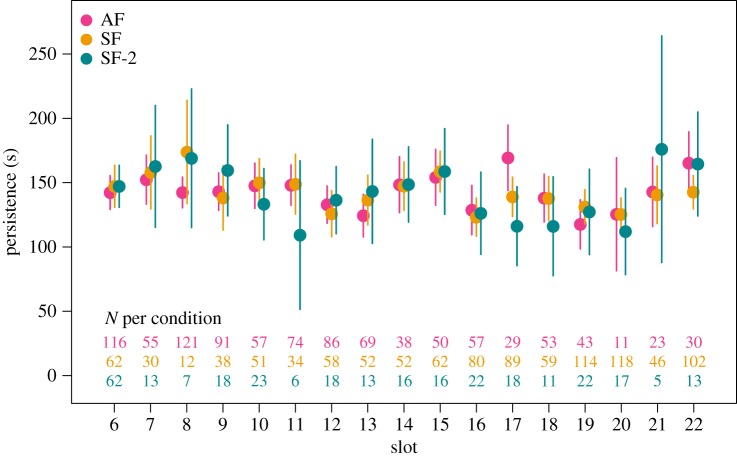
Persistence in the anagram-solving task when it was conducted before (AF), after (SF) or immediately after (SF-2) the Stroop task in slots 6–22. Numbers in the lower part of the figure denote the total number of participants included in each condition. Error bars denote 95% confidence intervals.

Figure 1 shows average persistence in the anagram-solving task. A multiple regression analysis with predictors slot (6–22) and condition (AF = +1 versus SF = −1) found a significant effect of slot, *b* = −0.667, s.e.*_b_* = 0.329, *t*_2059_ = −2.025, *p* = 0.043, but not condition, *b* = 0.266, s.e.*_b_* = 1.635, *t*_2059_ = 0.163, *p* = 0.871. That is, overall, participants who were asked to perform the anagram task relatively late within the experimental session tended to persist less, but there is no evidence to support the ‘postdiction’ that the length of time performing the anagram task would be shorter following the Stroop task.

For some participants, there were many tasks between the anagram and the Stroop task, and it is possible that any depletion caused by the Stroop task may have diminished over time. In that case, we would not expect to see evidence of depletion in these participants and putting them in the analysis might mask an effect that exists among other participants. To address this possibility, we compared participants in condition AF with a subset of participants in condition SF who had completed the anagrams immediately after the Stroop task (condition SF-2). A multiple regression analysis with predictors slot (6–22) and condition (AF = +1 versus SF-2 = −1) failed to detect a significant effect of either factor, *b* = −0.555, s.e.*_b_* = 0.404, *t*_1300_ = −1.372, *p* = 0.170, and *b* = 0.421, s.e.*_b_* = 2.253, *t*_1300_ = 0.187, *p* = 0.852, respectively.

**Figure 2.**
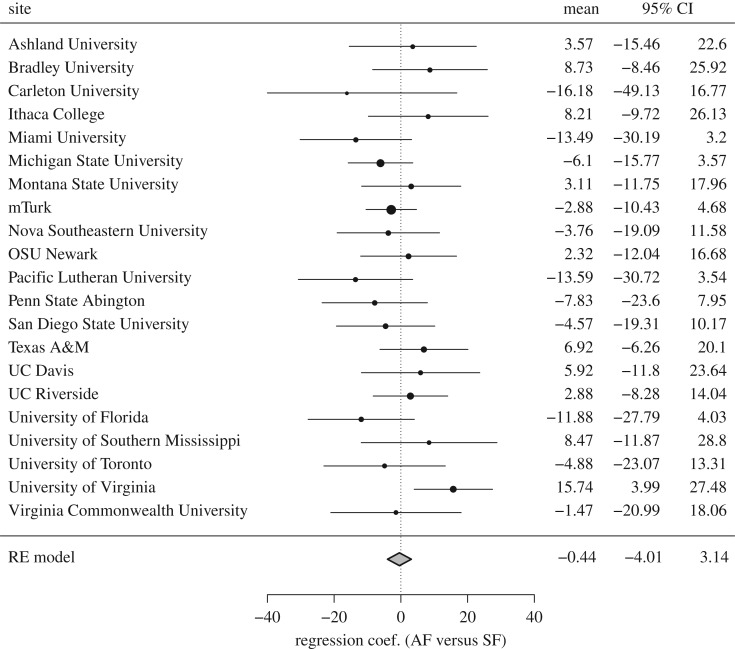
Forest plot and meta-analytic average of regression coefficients for the AF versus SF comparison across sites. Error bars denote 95% confidence intervals. The size of each data point represents its weight in the random-effects meta-analytic model shown in the lower row.

The primary data were gathered independently by 20 teams of researchers, including a sample of participants tested on mTurk. Given this variability, it is possible that the effect is still present in some subsamples. To explore this possibility, we conducted multiple regressions with factors slot and condition (AF = +1 versus SF = −1) independently for each subsample. Figure 2 shows the 95% confidence intervals of the regression coefficient for condition in each subsample. The final row represents the meta-analytic average, computed using a random effects model. Consistent with our previous analyses, we failed to detect a significant average effect of condition, *b* = −0.435, s.e.*_b_* = 1.822, 95% CI (−4.006, 3.136), *z* = −0.239, *p* = 0.811. The amount of heterogeneity across samples failed to reach statistical significance, *Q*_20_ = 23.326, *p* = 0.273, *I*^2^ = 19.07%, suggesting that differences across subsamples are no larger than would be expected by chance alone. The pattern of results does not change if the condition factor is recoded to compare the AF and SF-2 conditions, *b* = −0.574, s.e.*_b_* = 2.185, 95% CI (−4.857, 3.709), *z* = −0.262, *p* = 0.793, *Q*_20_ = 16.513, *p* = 0.684, *I*^2^ = 0%.

As can be seen in figure 2, only the sample recruited at the University of Virginia produced a regression coefficient significantly larger than zero, *b* = 15.736, s.e.*_b_* = 5.994, *t*_114_ = 2.625, *p* = 0.0098. Note that this *p*-value does not remain significant after applying the Bonferroni correction for multiple comparisons, *α* = 0.05/21 = 0.0024.

## 4. Discussion

In the present study, our objective was to examine evidence for ego depletion in the large dataset gathered for the Many Labs 3 project. Overall, our analyses failed to yield supportive evidence for the effect: participants who had completed the Stroop task did not persist any less in the anagram-solving task than the rest of the participants.

Importantly, this null result cannot be attributed to a lack of statistical power. The meta-analysis conducted by Hagger *et al*. [3] included 13 ego depletion studies that used the Stroop as the depleting task and they yielded an average effect size (Cohen's *d*) of 0.40, 95% CI (0.26, 0.55). This figure is highly consistent with the results of a recent meta-analysis by Dang [9], where the average effect among six studies using the Stroop was *g* = 0.44, 95% CI (0.18, 0.69). With 1003 participants in condition AF and 1059 in SF, our analysis had 0.983 power to detect an effect at the lower bound of the 95% confidence interval reported by Dang [9] for experiments specifically using the Stroop task, i.e. *g* = 0.18. Our analysis is also highly powered with respect to the impossible anagram-solving task. Carter *et al*. [8] found a meta-analytic effect size of 0.46, 95% CI (0.23, 0.69) among 21 studies using persistence in impossible anagram-solving tasks as the dependent variable. Our statistical power to detect an effect size at the lower bound of that confidence interval is 0.999. Therefore, even assuming that previous meta-analyses have grossly overestimated the size of the ego depletion effect observed with these tasks, our analyses should still have returned positive results. Of course, the previous power calculations rely on the assumption that the effect size estimations reported in previous meta-analyses are accurate and unbiased. If, as suggested by Carter *et al*. [7,8], this literature is strongly biased by the selective publication of significant findings, then the statistical power achieved in our analyses might not be as impressive as suggested by the figures above.

Compared to other experiments, the Stroop task implemented in Many Labs 3 was relatively brief and, unlike other studies using it as a depleting task, it included both consistent and inconsistent trials. It is possible that our failure to detect ego depletion is due to this feature of the procedure. However, we think that this is unlikely, as the effect size of the Stroop in Many Labs 3 was quite large by statistical standards (*d* = 0.91). Furthermore, the Stroop task was substantially longer in Many Labs 3 than in other studies that have used it as a depleting task [19] or as a secondary task [20] in previous ego depletion research.

Taken together with the negative results of Hagger *et al*. [10] and the clear evidence of publication bias or other reporting biases in this literature [7,8], the present results converge on the conclusion that ego depletion may not be a real effect (for a discussion, see [6]). Alternatively, it is possible that the effect is real, but contrary to previous research, either the Stroop is an ineffective means of depleting participants or persistence in an anagram-solving task is not a sensitive-dependent measure for detecting a depleting effect.

One thing that struck us during the analysis of the present dataset is that persistence was barely sensitive to simple fatigue effects: although persistence tended to decrease during the experimental session, the correlation between persistence and block was close to zero (*r* = −0.0493, *p* = 0.025). This is a very small effect, which only reaches statistical significance due to the large number of participants. If persistence turns out to be rather insensitive to general fatigue effects, it might also be insensitive to related effects like ego depletion.

**Figure 3.**
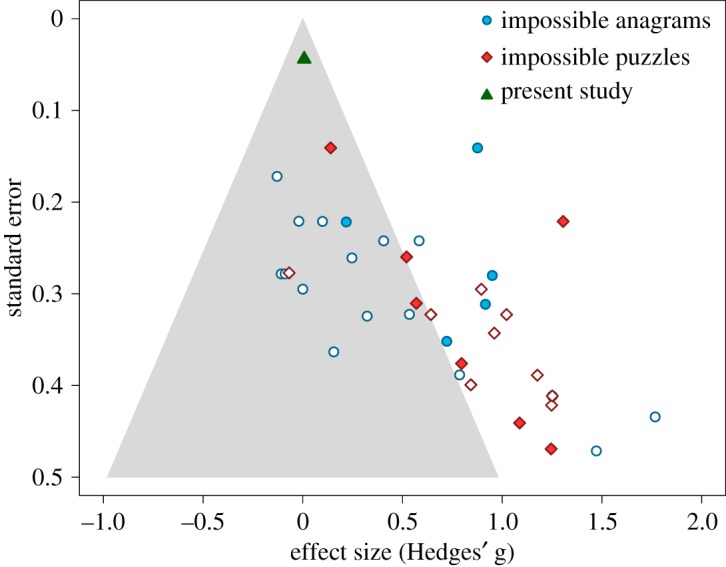
Funnel plot with effect sizes and standard errors meta-analysed by Carter *et al*. [8]. Filled circles and diamonds denote published studies, while white symbols denote unpublished studies. Effect sizes falling in the grey area are statistically non-significant in a two-tailed test with *α* = 0.05.

This interpretation casts doubts on the reliability of previous demonstrations of ego depletion using persistence in anagram solving as the main dependent variable. This concern is consistent with evidence from the meta-analysis conducted by Carter *et al*. [8], which included a subset of ego depletion studies that used impossible anagram solving as the dependent task and contained a funnel-plot analysis of the relationship between effect sizes and standard errors. In figure 3, we replot the effect sizes of those studies against their standard errors. We have also added effect sizes from a group of studies relying on a similar dependent measure (time invested in solving impossible puzzles) and the effect size of the present study. As can be seen, many of these effect sizes are statistically significant (i.e. they fall outside the grey area). However, this tends to happen more often for studies with low precision (i.e. higher standard error) than for studies with larger samples. With some caveats [21], this anomalous distribution of effect sizes is typically considered suggestive of publication or reporting biases. This means that the effect sizes reported in previous studies using this procedure might be a gross overestimation of the true effect sizes. If these effects are real, they might be too small to be detected even with very large samples comprising thousands of participants.

Of course, an alternative explanation of our failure to observe ego depletion in Many Labs 3 is that perhaps participants were not sufficiently motivated to try hard on the anagram-solving task, especially given that fact that, unlike standard ego depletion studies, Many Labs 3 relied on a rather long and probably tiresome experimental protocol. Note, however, that this explanation predicts that, if anything, ego depletion should have been observed at early stages of the experimental session and then vanished as participants became tired or lost motivation. The data depicted in figure 1 do not support this prediction.

In summary, the present study failed to find an ego depletion effect using a Stroop task as the depleting task and persistence in solving impossible anagrams as the dependent task. The lack of a meaningful fatigue effect on the impossible anagram task, together with the relationship between effect size and standard errors in meta-analysis of Carter *et al*. [8], suggests that persistence in impossible anagrams or puzzles might not provide a sensitive measure of ego depletion. Alternatively, it is possible that ego depletion is not a real effect, and that previous demonstrations of the effect are entirely due to publication biases, questionable research practices or other artefacts.

## Data accessibility

The complete dataset of Many Labs 3 is available at https://osf.io/tdgk9/. The scripts used in the present analyses are available at https://osf.io/852ne/.
